# From Epidemic Meningitis Vaccines for Africa to the Meningitis Vaccine Project

**DOI:** 10.1093/cid/civ593

**Published:** 2015-11-09

**Authors:** M. Teresa Aguado, Luis Jodar, Dan Granoff, Regina Rabinovich, Costante Ceccarini, Gordon W. Perkin

**Affiliations:** 1 Vaccines and Immunization Consultant, Versoix, Switzerland; 2 Pfizer Inc, New York City, New York; 3 Children's Hospital Oakland Research Institute, California; 4 Harvard School of Public Health, Boston, Massachusetts; 5 Vaccine Consultant, Siena, Italy; 6 Public Health Consultant, Seattle, Washington

**Keywords:** meningitis epidemics, group A meningococcal conjugate vaccines, feasibility study, innovation, public-private partnership

## Abstract

***Background.*** Polysaccharide vaccines had been used to control African meningitis epidemics for >30 years but with little or modest success, largely because of logistical problems in the implementation of reactive vaccination campaigns that are begun after epidemics are under way. After the major group A meningococcal meningitis epidemics in 1996–1997 (250 000 cases and 25 000 deaths), African ministers of health declared the prevention of meningitis a high priority and asked the World Health Organization (WHO) for help in developing better immunization strategies to eliminate meningitis epidemics in Africa.

***Methods.*** WHO accepted the challenge and created a project called Epidemic Meningitis Vaccines for Africa (EVA) that served as an organizational framework for external consultants, PATH, the US Centers for Disease Control and Prevention (CDC), and the Bill & Melinda Gates Foundation (BMGF). Consultations were initiated with major vaccine manufacturers. EVA commissioned a costing study/business plan for the development of new group A or A/C conjugate vaccines and explored the feasibility of developing these products as a public–private partnership. Representatives from African countries were consulted. They confirmed that the development of conjugate vaccines was a priority and provided information on preferred product characteristics. In parallel, a strategy for successful introduction was also anticipated and discussed.

***Results.*** The expert consultations recommended that a group A meningococcal conjugate vaccine be developed and introduced into the African meningitis belt. The results of the costing study indicated that the “cost of goods” to develop a group A – containing conjugate vaccine in the United States would be in the range of US$0.35–$1.35 per dose, depending on composition (A vs A/C), number of doses/vials, and presentation. Following an invitation from BMGF, a proposal was submitted in the spring of 2001.

***Conclusions.*** In June 2001, BMGF awarded a grant of US$70 million to create the Meningitis Vaccine Project (MVP) as a partnership between PATH and WHO, with the specific goal of developing an affordable MenA conjugate vaccine to eliminate MenA meningitis epidemics in Africa. EVA is an example of the use of WHO as an important convening instrument to facilitate new approaches to address major public health problems.

The history of epidemic meningitis in sub-Saharan African countries has been well documented [1]. Every year from January to May when much of sub-Saharan Africa is dry, windy, and dusty, epidemics of meningococcal meningitis occur. Every 10–15 years, meningococcal epidemics can assume major proportions such as the 1996 epidemic, which caused an estimated 250 000 cases with >25 000 deaths [2].

Until the recent introduction of a monovalent group A meningococcal conjugate vaccine, PsA-TT (MenAfriVac), group A *Neisseria meningitidis* accounted for about 80%–85% of cases [3]. Meningitis is one of the most feared diseases on the African continent. Meningitis epidemics caused major disruptions of fragile national public health systems because of the severity of the illnesses and the geographic unpredictability of where the epidemics would occur. In addition to a case fatality rate of about 10%, approximately 20% of survivors suffer serious sequelae such as deafness, mental retardation, and seizures.

Polysaccharide (PS) A/C vaccines had been available for 20 years, and their use in reactive campaigns that are begun in response to epidemics was strongly encouraged by the World Health Organization (WHO) [4, 5]. The WHO International Coordinating Group was created in 1997 to facilitate the acquisition and shipment of meningitis vaccines to African countries during meningitis epidemics. The International Coordinating Group also facilitated development of national preparedness plans and response through its technical partners and their networks.

There were, however, important logistical problems in implementation of this reactive strategy. Epidemics had to be detected early and the etiologic agent identified. In the 1990s, basic microbiological capabilities were in short supply in sub-Saharan countries, and there was often a delay between identification of an outbreak and determining the agent responsible for the epidemic. Funds for vaccine purchases had to be mobilized, and supplies of PS vaccines had to be identified, purchased, and shipped to epidemic countries. Country resources had to be in place so vaccines could be properly received, stored, and distributed to epidemic areas where vaccinations were done. Each of these steps took time, such that meningococcal PS vaccines were often given after the epidemics were over. The end result was that most of the reactive campaigns yielded only marginal public health benefit.

In addition, meningococcal group A/C PS vaccines were not considered to be optimal products for routine vaccination programs. Group A/C PS vaccines were not recommended for children <24 months of age and did not induce long-term memory. The PS vaccines did not decrease colonization and would not be expected to generate herd protection [5].

## DEVELOPMENT OF THE EPIDEMIC MENINGITIS VACCINES FOR AFRICA PROJECT

After the catastrophic 1996 epidemic, a meeting of 26 ministries of health (MOH) from meningitis belt countries (Benin, Burkina Faso, Burundi, Cameroon, Central African Republic, Chad, Côte d'Ivoire, Democratic Republic of Congo, Ethiopia, Erithrea, Ghana, Guinea, Guinea-Bissau, Kenya, Mali, Mauritania, Niger, Nigeria, Rwanda, Senegal, South Sudan, Sudan, Tanzania, The Gambia, Togo, and Uganda) took place in Ouagadougou, Burkina Faso. MOH delegates recognized that epidemic meningitis was a public health emergency and that the interventions being used were inefficient, so countries committed themselves to work with WHO and to shift their strategies from epidemic response to epidemic preparedness [5].

WHO's Immunization, Vaccines and Biologicals department began addressing this challenge in 1997 and 1998 by supporting in part the clinical development of the group A/C conjugate vaccines as well as reviewing with vaccine manufacturers their plans for developing group A conjugate vaccines. In addition, there was great hope that an effort to develop a group C meningococcal conjugate vaccine might include a group A conjugate vaccine as well, thus creating a potentially valuable product for African meningitis belt countries. In the end, and disappointingly, it was chosen to pursue the development of monovalent group C conjugate vaccines. A group A–containing vaccine only for the sub-Saharan countries was not considered a market driver.

In an effort to formalize the African meningitis vaccine work, WHO created the Epidemic Meningitis Vaccines for Africa (EVA) project, led by Teresa Aguado and Luis Jodar. They sought help from Dan Granoff from the Children's Hospital Oakland Research Institute (California) and Nancy Messonier from the US Centers for Disease Control and Prevention (CDC) in Atlanta, Georgia. Together they worked to codify the rationale and goals of the EVA project as follows:

1. To prevent and ultimately eliminate meningococcal epidemics in the African meningitis belt, a new vaccine should (1) be immunogenic in infants and induce long-term protection in all age groups and (2) decrease nasopharyngeal carriage and transmission, thereby providing herd protection. Herd protection was considered to be of particular value in preventing meningococcal epidemics in the countries of the African meningitis belt where infant immunization rates at that time were <50%.
2. Conjugate vaccines could eliminate meningococcal epidemics in the meningitis belt. Other conjugate vaccines had been highly successful in preventing *Haemophilus influenzae* type b (Hib) and pneumococcal infections. They were safe and effective in infants and older children. Immunization also decreased nasopharyngeal colonization and transmission of the organism [6]. The new conjugate vaccines showed great promise because these vaccines initiated a T-cell response with heightened immunogenicity and could be used in toddlers aged <2 years, an age group in which polysaccharide vaccines are ineffectual. The meningococcal conjugate vaccines tested to that date had similar immunologic properties as Hib and pneumococcal conjugates and were predicted to be equally effective [7].
3. Group C meningococcal conjugate vaccines were licensed in Europe and first used in the United Kingdom in 1999 without phase 3 efficacy study, and had been introduced from infants to young adults. Data indicated that these vaccines were safe and highly effective in decreasing the incidence of group C disease in the United Kingdom [8]. Serum bactericidal antibodies were a generally accepted surrogate of protection against meningococcal disease. Therefore, the availability of a serologic surrogate of efficacy would provide a reliable early milestone for assessing the likelihood of success of a meningococcal conjugate vaccine for Africa.
4. Group A/C meningococcal conjugate vaccines had been tested in African infants. In Niger, a WHO/CDC/Centre de Recherche Médicale et Sanitaire project showed that these vaccines were well tolerated and immunogenic, eliciting high titers of bactericidal antibody [9]. In The Gambia, one conjugate vaccine showed induction of immunologic memory for the C component, important for long-term protection; however, the A component behaved differently depending on the populations tested [10].
5. Intellectual property rights for the conjugation process were in the public domain.
6. Technology and know-how were available from several manufacturers and laboratories.
7. For more than a decade, several manufacturers had developed and clinically evaluated group A/C meningococcal conjugate vaccines. However, at the time of initiation of the EVA project, all of these development programs had stopped. Possible reasons were:
  - Lack of market potential. Group A meningococcus was virtually nonexistent in industrialized countries, and disease caused by this strain was limited to Africa and some areas in the Eastern Mediterranean region and Asia.
  - Research and development, clinical, and licensing costs were not justified by the expected low return on investment. Opportunity costs were also cited—for example, that producing a vaccine of low commercial value could conflict with other projects perceived to be more commercially valuable.
  - Existing manufacturing capacity for conjugate vaccines was insufficient to cope with the required number of doses.
  - Several manufacturers were in the process of producing either group C or polyvalent vaccines for global use.
8. In addition, the timing for the development of new meningococcal vaccines was perceived as appropriate. The Global Alliance for Vaccines and Immunization (Gavi) had recently been launched, and the Bill & Melinda Gates Foundation (BMGF) and other partners were injecting new resources into global health. Last, there was interest in stimulating the development of needed vaccines in the developing world through new pull-and-push mechanisms.
9. Finally, WHO believed that there was an opportunity to involve a number of partners such as the CDC, Agence de Médecine Préventive, Médecins Sans Frontières, and UNICEF with specific expertise in epidemiology, vaccine evaluation, epidemic control, and immunization program management. These partners had already collaborated with WHO in specific projects, and their expertise could contribute to the quality of the effort.

WHO, through the International Federation of Pharmaceutical Manufacturers and Associations, initiated discussions with 5 large international vaccine producers. Three manufacturers expressed interest in a possible future collaboration with WHO for the development of a group A conjugate vaccine to be used in Africa.

EVA commissioned a study that would explore manufacturing costs to make a MenA conjugate vaccine. The proposal (a business plan exploring different alternatives for public-private partnerships for the development of a group A meningococcal conjugate vaccine) described a not-for-profit company (NFP) for the development of a group A conjugate vaccine. The proposal included 3 approaches for a public–private partnership: (1) an alliance with a major vaccine company; (2) manufacturing, in part, at the NFP; and (3) establishment of a full manufacturing facility at the NFP. Each of the options looked at feasibility, resources required, timelines for licensure, and ultimate costs of producing a group A or group A/C meningococcal conjugate vaccine. The results of the exercise showed that a US vaccine manufacturer making those vaccines at volumes of 25–50 million doses annually could do so at a cost of US$0.35–$1.35 per dose, depending on composition, number of doses/vials, and type of formulation (liquid or lyophilized). The model included financing costs incurred in building a vaccine manufacturing plant and a fill/finish facility to package the vaccine. The facility was depreciated over 10 years, and clinical and regulatory costs were also included. Therefore, if facilities and fill/finish lines were already available, a monovalent group A conjugate vaccine could be made for as low as US$0.20 per dose as long as annual volumes were >25 million doses.

This feasibility study provided EVA with a credible framework for discussions with vaccine manufacturers when evaluating options in the creation of a public–private venture to produce the vaccine. In addition, it provided a blueprint for alternative public–private partnerships for other developing-country market vaccines. Seed funding to support these and other related activities was provided in part by the US Agency for International Development.

An introduction plan for the new vaccine was also designed and consisted of a routine immunization schedule for infants <1 year of age and mass immunization campaigns for those 1–29 years of age. Different immunization schedules were contemplated, and a phased introduction including a pilot effectiveness study was also considered.

The EVA project was presented on 5–7 April 2000 at a WHO meeting titled “Conjugate Vaccines Against Meningococcal Disease in the African and Eastern Mediterranean Region,” during which the 3 pillars of the project were presented and discussed: (1) business plan and feasibility study; (2) strategy for introduction; and (3) ensuring appropriate advocacy and political commitment. Following this consultation, an AFRO/EMRO (WHO's regional offices in the African and Eastern Mediterranean regions, respectively) statement was drafted that endorsed the development of a new and affordable group A conjugate vaccine. The delegates made several recommendations, including (1) giving priority to developing a group A vs an A/C conjugate vaccine; (2) indicating a preference for a single-dose schedule; (3) favoring introduction through mass campaigns and into the routine Expanded Programme on Immunization (EPI) schedule; and (4) strengthening meningitis surveillance [11].

In June 2000, EVA was asked to present its project to BMGF in Seattle. The meeting compared and debated strategies to effectively fight the African meningitis epidemics. Experts vigorously debated the advantages and constraints of expanding the use of the existing PS vaccines vs a new group A or A/C meningococcal conjugate vaccine aimed at eliminating epidemics in African countries.

## DEVELOPMENT OF THE MENINGITIS VACCINE PROJECT: A COLLABORATION BETWEEN WHO AND PATH

For EVA's ambitious goals to be realized, a core group with expertise in vaccine development and public–private partnerships was needed. In July 2000, EVA initiated discussions with PATH, a Seattle-based nongovernmental organization with technical, legal, and operational experience in the development of needed products for resource-poor countries. The PATH team was led by Regina Rabinovich, who provided expertise and experience in the creation of public–private partnerships that addressed business as well as technical challenges. PATH and WHO worked together on a product development plan that included a private-sector vaccine producer partner, with technical support from key stakeholders such as the CDC. In addition, multiple meetings and consultations with a variety of experts were held in which strategies were dissected.

In February 2001, a proposal was sent to BMGF requesting funding for a 10-year project at the level of US$70 million. In May 2001, WHO and PATH were informed that a US$70 million grant was approved, and a public announcement was made in Washington, D.C. by Patty Stonesifer, then the president of BMGF, in the presence of representatives of both institutions. EVA had now evolved into the Meningitis Vaccine Project (MVP).

Key to the success of the collaboration was the conjunction of several elements: (1) a sound project, with needs and solutions clearly understood and supported by solid scientific evidence; (2) an optimal combination of several partners with different but complementary strengths; and (3) the financial support at a scope to make success possible. The grant provided core funding for the vaccine development through licensure and postlicensure demonstration projects, with the understanding that funds for vaccine purchases would be sought elsewhere.

## DISCUSSION

The EVA project proved to be unique and effective in addressing an important public health problem. The following hallmarks are worth noting:

1. It originated because of a pressing public health need that had a sound rationale for resolving the problem.
2. Other conjugate vaccines had a proven record of success, and more specifically, group C meningococcal conjugate vaccine introduction was proving to be highly effective in the United Kingdom.
3. The solutions proposed dealt in parallel with diverse issues ranging from research and development through scaling up and production, quality, supply, and financing to large-scale vaccine introduction at national level.
4. EVA anticipated the needs for a proper introduction in sub-Saharan Africa, one of the most difficult areas of the world for the delivery of immunization programs. For example, the need for significantly improved meningitis surveillance in the region was recognized as a priority.
5. The commissioned feasibility study provided the project with a well-informed basis and a credible framework for discussions with manufacturers.
6. From the outset, EVA solicited political commitment, support, and endorsement of the end users—the countries in need of the vaccine—who requested not only the right product, but an affordable one.
7. EVA, and subsequently MVP, offered a good model that could be replicated for other developing country market vaccines [12]
8. The size of the award was commensurate with the funding required for such an ambitious project. This allowed the project team to drive the MVP development process without the interruption of continuously seeking funds for the core effort.

The EVA project involved a number of stakeholders, including several WHO departments, meningitis experts, vaccine producers, and public health institutions, some of whom continued with MVP. EVA insisted on documentation and analysis on one hand, and the projection of different scenarios to reach the goals on the other. The PATH/WHO partnership resulted in great complementarity.

EVA offered a venue for all agencies that were working on African meningitis, where all ideas were considered in an open environment which, in the end, resulted in robust funding for MVP.

Eventually, MVP's development work led to approaching a broader group of vaccine manufacturers from developing countries who were interested in making a new meningococcal vaccine for Africa. Among these new manufacturers, the Serum Institute of India, Ltd (SIIL) was selected in 2003 to manufacture the new vaccine that was licensed by Indian regulatory authorities in December 2009 and prequalified by WHO in 2010 [13, 14]. To date, approximately 210 million doses of group A meningococcal conjugate vaccine manufactured by SIIL, MenAfriVac, have been distributed in well-implemented campaigns in the target countries.

The progression from WHO's EVA to the MVP partnership between PATH and WHO is a useful example of the key convening role that WHO can play in serving as an organizing locus to allow for creative discussions that led to the formulation of a comprehensive solution to an important public health problem. When considered in retrospect, it was an elegant example of partnership: at the same time that WHO was key in pulling partners together, PATH was key in the preparation of a more comprehensive grant to BMGF through its understanding and experience developing new products, while the CDC and other partners provided technical expertise. Success in obtaining support from BMGF occurred because the foundation recognized the quality of the partnership that was being proposed. In the end, the scope of BMGF support offered the newly created project, MVP, the opportunity to succeed.

EVA and MVP represented a big challenge, but they also offered to all involved a unique opportunity to achieve major impact with the resulting product, a new vaccine, and a model to follow for other undertakings.
